# Toward Integrated Models of Infectious Diseases, Harm Reduction, and Primary Care

**DOI:** 10.1093/ofid/ofaf484

**Published:** 2025-08-12

**Authors:** Suhanee Mitragotri, Aakash Reddy, Kevan Shah, David T Zhu

**Affiliations:** Harvard College, Harvard University, Cambridge, Massachusetts, USA; Weill Cornell Medical College, New York, New York, USA; Cooper Medical School of Rowan University, Camden, New Jersey, USA; Medical Scientist Training Program, School of Medicine, Virginia Commonwealth University, Richmond, Virginia, USA

To the  Editor—Knodle et al conducted a retrospective observational study of a pilot program in Chicago, Illinois, that integrated primary care and infectious disease services within a community-based harm reduction setting [1]. Between 2018 and 2021, the program served 552 patients, with the majority of visits addressing substance use disorders (SUD) and related infections, such as hepatitis C and HIV. The study found that incorporating trusted providers into harm reduction programs led to increased treatment engagement among people who use drugs (PWUD), a population often marginalized from traditional healthcare due to stigma, structural exclusion, and fear of criminalization. This model of colocated care reflects a core component of street medicine and demonstrates how integrated care is an equity-centered framework for delivering low-threshold addiction and harm reduction care.

Two key elements of this model are noteworthy. First, the central role of outreach workers with lived SUD experiences likely contributed to increased patient engagement. These peer workers serve as cultural bridges between healthcare systems and communities, fostering trust that traditional models often struggle to establish. Second, bundling multiple services into a single access point precludes PWUD from having to navigate traditionally fragmented healthcare systems. Prior evidence suggests that such integrated models reduce patient attrition, increase patient engagement, and lower substance use relapse rates [2].

However, resource availability represents a significant barrier to the widespread implementation of these integrated models. Many community-based harm reduction programs operate on limited budgets and may lack the physical space or infrastructure required to provide comprehensive care. Additionally, harm reduction programs may struggle to recruit and retain qualified healthcare providers willing to work in these settings because of factors such as stigma associated with PWUD [3]. Regulatory hurdles on syringe services programs in many jurisdictions further constrain implementation as some states lack explicit laws that authorize syringe services programs or the free distribution of syringes [4].

To overcome these obstacles, targeted policy reforms are needed. The precarious, often grant-reliant funding that characterizes many community-based programs precludes the long-term sustainability of these programs. First, as harm reduction programs begin integrating clinical services, traditional fee-for-service models prove inadequate for reimbursing the time-intensive, multidisciplinary care these settings provide. One alternative to explore includes shared-savings models, which would enable integrated harm reduction programs to benefit from the downstream cost reduction they generate [5]. Most critically, payment structures should accurately reflect the complex medical and social needs of PWUD, including high levels of comorbid mental illness and housing instability.

Second, workforce solutions must be designed to reflect the clinical and cultural competencies necessary for effective care delivery. This includes expanding eligibility for federal loan repayment programs, such as the National Health Service Corps (NHSC), to providers in harm reduction settings. A recent study found that an expansion of the NHSC loan repayment programs increased the number of clinicians providing behavioral health services and the provision of substance use disorder treatment in underserved areas [6]. Moreover, federal investment through agencies such as the Health Resources and Services Administration could fund the training and formal integration of peer outreach workers, recognizing their key role in patient engagement and retention.

Third, states have a responsibility to drive innovation and can use Medicaid waivers to pilot and scale integrated care models. Medicaid agencies should be encouraged to create streamlined pathways for harm reduction centers to serve as designated care hubs for PWUD. The recent establishment of CMS Place-of-Service Code 27 for street medicine sets a clear precedent; analogous billing codes for comprehensive harm reduction clinics would allow states to formally certify these sites and provide reimbursement for the full scope of services they deliver [7].

The model presented by Knodle et al is more than a promising pilot; it demonstrates that with thoughtful design and trusted community partnerships, we can effectively engage PWUD in comprehensive care. However, to move from isolated successes to systemic solutions, we must champion robust policy changes. By adopting sustainable funding structures and investing in a dedicated workforce, we can create an environment where integrated harm reduction programs can thrive. Such investments are crucial for reducing long-term healthcare expenditures and advancing public health.
